# Recent Advances in Polyphenylene Sulfide-Based Separators for Lithium-Ion Batteries

**DOI:** 10.3390/polym17091237

**Published:** 2025-04-30

**Authors:** Lianlu Wan, Haitao Zhou, Haiyun Zhou, Jie Gu, Chen Wang, Quan Liao, Hongquan Gao, Jianchun Wu, Xiangdong Huo

**Affiliations:** 1 School of Materials Science and Engineering, Jiangsu University, Zhenjiang 212013, China; 2212205082@stmail.ujs.edu.cn (L.W.); 2212305007@stmail.ujs.edu.cn (J.G.); 2222405033@stmail.ujs.edu.cn (C.W.); 2222205001@stmail.ujs.edu.cn (Q.L.); 1000004675@ujs.edu.cn (H.G.); jcwu@ujs.edu.cn (J.W.); 2 Jiangsu Tongling Electric Co., Ltd., Zhenjiang 212200, China

**Keywords:** polyphenylene sulfide (PPS) separator, high-energy-density batteries, dry-film process, thermal stability, structure–property relations

## Abstract

Polyphenylene sulfide (PPS)-based separators have garnered significant attention as high-performance components for next-generation lithium-ion batteries (LIBs), driven by their exceptional thermal stability (>260 °C), chemical inertness, and mechanical durability. This review comprehensively examines advances in PPS separator design, focusing on two structurally distinct categories: porous separators engineered via wet-chemical methods (e.g., melt-blown spinning, electrospinning, thermally induced phase separation) and nonporous solid-state separators fabricated through solvent-free dry-film processes. Porous variants, typified by submicron pore architectures (<1 μm), enable electrolyte-mediated ion transport with ionic conductivities up to >1 mS·cm^−1^ at >55% porosity, while their nonporous counterparts leverage crystalline sulfur-atom alignment and trace electrolyte infiltration to establish solid–liquid biphasic conduction pathways, achieving ion transference numbers >0.8 and homogenized lithium flux. Dry-processed solid-state PPS separators demonstrate unparalleled thermal dimensional stability (<2% shrinkage at 280 °C) and mitigate dendrite propagation through uniform electric field distribution, as evidenced by COMSOL simulations showing stable Li deposition under Cu particle contamination. Despite these advancements, challenges persist in reconciling thickness constraints (<25 μm) with mechanical robustness, scaling solvent-free manufacturing, and reducing costs. Innovations in ultra-thin formats (<20 μm) with self-healing polymer networks, coupled with compatibility extensions to sodium/zinc-ion systems, are identified as critical pathways for advancing PPS separators. By addressing these challenges, PPS-based architectures hold transformative potential for enabling high-energy-density (>500 Wh·kg^−1^), intrinsically safe energy storage systems, particularly in applications demanding extreme operational reliability such as electric vehicles and grid-scale storage.

## 1. Introduction

With the continuous advancement of global industrialization, the demand for energy is constantly increasing. Traditional fossil fuels such as oil, coal, and natural gas are being depleted at an accelerating rate due to their non-renewable nature [1,2]. Consequently, achieving green and sustainable energy storage solutions has become a shared goal among researchers. Lithium-ion batteries (LIBs) have emerged as one of the most effective and promising options among various energy storage systems due to their high energy density [3,4,5], high power density [6,7,8], low self-discharge [9,10,11], and long cycle life [12,13]. These attributes render them exceptionally well-suited for diverse applications, encompassing portable electronic apparatus, electrified transportation systems, and intelligent power grids [14,15,16].

In recent decades, the proposal of low-carbon economy goals has spurred the accelerated evolution of lithium-ion battery technologies, while also imposing higher requirements on each component of these batteries [17,18]. Traditional lithium-ion batteries primarily consist of four parts: the anode, cathode, separator, and electrolyte [19]. During the electrochemical charging phase, lithium ions migrate from the cathode through the electrolyte and separator to the anode, where they are embedded. Conversely, throughout the electrochemical discharge phase, lithium ions move from the anode through the electrolyte and separator back to the cathode, where they re-embed [20]. Despite the separator’s non-reactive nature in the cell’s electrochemical processes, it plays a crucial role by physically separating the anode and cathode. It prevents internal electron transfer, avoids internal short circuits, and facilitates the orderly transport of lithium ions, making it an indispensable component of lithium-ion batteries [21]. The structure and properties of the separator significantly impact the internal resistance, safety, cycling stability, and capacity of the battery [22]. An ideal separator for lithium-based batteries should exhibit low internal resistance, excellent chemical stability, high porosity, superior wettability, high ionic conductivity, strong mechanical properties, and high thermal stability [23,24].

Microporous polyolefin separators have achieved commercialization and are widely used in lithium-ion batteries due to their high electrochemical stability, excellent mechanical strength, and outstanding cost-effectiveness [25,26,27]. However, non-polar polyolefin separators exhibit poor wettability with carbonate-based liquid electrolytes, leading to low ionic conductivity and high internal resistance. Additionally, the inherently low melting points of polyolefins (approximately 135 °C for PE and PP) result in poor thermal stability. Under high temperatures or heavy loads, these separators are prone to thermal shrinkage, which can cause short circuits between the electrodes, drastically impeding their deployment in high-capacity energy storage configurations [28,29,30,31]. Furthermore, the inadequate interfacial contact between polyolefin-based separators and electrode materials increases the risk of uneven lithium dendrite growth. The large pores and low modulus of polyolefin separators make them ineffective in suppressing lithium dendrite formation, substantially diminishing the electrochemical cycling stability and compromising its operational safety [32,33,34,35,36]. Therefore, developing novel separators to address these challenges is of paramount importance, aiming to provide safer and more efficient energy storage solutions.

Polyphenylene sulfide (PPS) represents a linear polymeric architecture characterized by alternating aromatic rings and sulfur linkages, demonstrating outstanding chemical inertness, exceptional thermal stability (with short-term heat resistance up to 260 °C and long-term usability at 200–240 °C), outstanding mechanical properties, superior electrical performance, and good processability. These superior characteristics have driven its pervasive implementation in numerous technological domains, such as environmental protection, automotive, electronics, and aerospace [37,38]. In comparison to conventional polyolefin-based materials, PPS demonstrates superior performance characteristics, most notably its outstanding thermal stability, evidenced by an exceptionally high limiting oxygen index (LOI) of 46%. The presence of sulfur atoms within its molecular architecture induces a synergistic effect that substantially augments the material’s inherent flame-retardant capabilities. During thermal decomposition, PPS undergoes a rapid carbonization process via radical-mediated reactions with bicyclic carbon structures, forming a compact char layer that effectively impedes thermal and oxidative diffusion, thereby exhibiting remarkable flame inhibition properties. This intrinsic self-extinguishing behavior results in immediate flame suppression upon ignition source removal, considerably enhancing operational safety parameters. Conversely, traditional polyolefin separators exhibit pronounced thermoplastic deformation under elevated temperatures, potentially causing electrode short-circuiting and subsequent thermal runaway events. With an autoignition temperature reaching 290 °C, PPS-based separators provide lithium-ion battery systems with significantly enhanced thermal protection capabilities. Moreover, highly crystalline polyphenylene sulfide (PPS) enables rapid transport of Li^+^, Na^+^, and Zn^2+^ ions through its sulfur-anchored ordered channels. Our previous research revealed [39] that ultrahigh-crystallinity (>60%) polyphenylene sulfide (PPS) dense membranes, as measured by pulsed field gradient solid-state nuclear magnetic resonance (PFG-SSNMR), exhibit an intrinsic lithium-ion diffusion coefficient (D_7Li_) of 10^−7^–10^−8^ cm^2^ s^−1^ at room temperature. This performance is comparable to that of sulfide-based solid electrolytes (e.g., (Li_2_S)_7_(P_2_S_5_)_3_, DLi = 4.58 × 10^−8^ cm^2^ s^−1^), suggesting their potential to achieve high room-temperature ionic conductivity (>0.001 S cm^−1^). Independent studies by Ionic Materials [40] and LG Energy Solution (LGES) [41] have validated the superior ionic conductivity of PPS-based solid-state electrolytes, demonstrating their potential as high-performance ion-conductive separators for next-generation batteries.

Given PPS’s excellent physical and chemical properties, researchers have explored its application in battery separators through various preparation methods, including melt-blown spinning, electrospinning, island-in-sea spinning, and dry rolling. In 2015, Wang et al. [42] proposed using PPS as a raw material to prepare nonwoven separators for lithium-ion batteries via melt-blown spinning technology, enhancing battery safety and extending cycle life. In 2019, Zhou et al. [43] employed a pilot-scale solvent-free process combined with high-speed air blowing and hot rolling to produce self-supporting films at relatively low temperatures, demonstrating excellent electrochemical performance and high safety. However, further practical research is needed to advance the use of these membranes in more reliable lithium battery systems, including lithium metal batteries (LMBs) and lithium metal-free anode batteries (LMFBs).

Throughout this investigation, we comprehensively review the latest advancements in the application of polyphenylene sulfide (PPS) separators in lithium-ion batteries. We first establish the core specifications governing lithium-ion battery separator materials. Next, we discuss the current preparation techniques used for the fabrication and modification of PPS separators. Finally, we focus on the specific applications of PPS separators in lithium-ion batteries, including lithium metal batteries (LMBs) and lithium metal-free anode batteries (LMFBs). Methodically, we rigorously analyze the fabrication techniques and performance characteristics of PPS membranes to establish their electrochemical functionality and the challenges they face when applied in energy storage systems. Furthermore, this article explores the future prospects and research directions for PPS separators in lithium-ion batteries, aiming to provide practical guidance and valuable insights to support the ongoing development and application of PPS separators in lithium-ion batteries.

## 2. The Basic Requirements for Lithium Battery Separators

As an essential constituent element in lithium-ion battery architectures, the separator serves the dual function of isolating the cathode and anode assemblies to mitigate internal shorting while facilitating unimpeded Li^+^ ion conduction. Separator failures (such as physicochemical degradation, thermal runaway, lithium dendrite growth, etc.) are among the primary causes of battery failure [44], necessitating stringent performance requirements [45]. First, the chemical and electrochemical stability of the separator is critical to battery lifespan. It must remain stable in highly oxidizing or reducing environments and withstand the corrosion from electrolytes and electrode materials [46]. A separator with strong electrochemical stability can endure higher voltages; ideally, the electrochemical window of a dry separator should exceed 0 V, with a potential higher than 5 V relative to the Li^+^/Li reference electrode. Second, the pore structure of the separator requires precise regulation. Pores that are too small can hinder lithium-ion transport, while pores that are too large may fail to effectively block electrode material penetration, thereby compromising battery safety [47]. Studies suggest that submicron-sized pores (<1 μm) are ideal [46], as they ensure efficient lithium-ion transport while significantly reducing the risk of short circuits [48,49,50]. Additionally, the uniformity of pore distribution is crucial. Non-uniform pore distribution can lead to imbalanced lithium-ion flow, triggering lithium dendrite growth and adversely affecting battery performance and lifespan [51,52,53]. Porosity is a key parameter determining lithium-ion transport efficiency, typically requiring a value above 40% to facilitate sufficient electrolyte infiltration and efficient lithium-ion diffusion. However, excessively high porosity can weaken the mechanical strength of the separator [54], thereby impacting battery safety. The wettability of the separator also significantly influences lithium-ion transport efficiency. Superior electrolyte affinity improves ion transport efficiency and diminishes interfacial impedance [55,56,57]. Lithium-ion conductivity is a vital metric for evaluating battery performance, closely related to the separator’s wettability. Generally, the ionic conductivity of an electrolyte-filled separator should not be less than 10^−3^ S/cm^2^ [58]. Higher conductivity not only reduces internal resistance but also effectively suppresses lithium dendrite growth, thereby improving battery safety. Separator dimensional profile exerts substantial influence on electrochemical stability and operational safety. The ideal thickness range is 20–25 μm. Thicker separators increase internal resistance and reduce energy density [59], while thinner separators compromise mechanical strength, affecting battery reliability. Furthermore, the separator must exhibit outstanding mechanical characteristics, encompassing tensile robustness and perforation resilience to withstand external forces and challenges posed by lithium dendrite growth [60]. Finally, the thermal robustness of the separator constitutes a critical determinant in mitigating battery thermal runaway [61]. It must maintain dimensional stability under high temperatures, typically requiring a shrinkage rate of less than 5% after 60 min at 90 °C to ensure safe battery operation.

## 3. Preparation Process of Polyphenylene Sulfide Separator

### 3.1. Nanofiber Nonwoven Fabric

In recent years, nanofiber nonwoven separators have attracted significant attention [62,63]. Their high porosity ensures excellent electrolyte uptake and superior ionic conductivity, while nonwoven separators made from high-temperature-resistant materials also help prevent battery thermal runaway, enhancing battery safety [64]. Over the past few decades, nonwoven separators prepared from materials such as polyacrylonitrile (PAN) [65,66,67,68], polyimide (PI) [69,70], polyvinylidene fluoride (PVDF) [71], and polyvinylidene fluoride–hexafluoropropylene (PVDF-HFP) [72,73] have been widely used in lithium-ion batteries. Polyphenylene sulfide (PPS) exhibits excellent chemical stability, exceptional thermal stability, outstanding mechanical properties, and superior electrical performance, making it a promising material for nonwoven separators in lithium-ion batteries [74]. Currently, the processes used for preparing PPS nanofiber nonwoven separators include melt-blown spinning, electrospinning, and island-in-sea spinning.

#### 3.1.1. Melt-Blown Spinning

Melt-blown spinning is a fiber-forming method in which a thermoplastic polymer melt is extruded and rapidly stretched and solidified by high-speed, high-temperature airflow. The key factor for using melt-blown spinning to prepare ultrafine fibers is the appropriate fluidity of the thermoplastic polymer melt. Polyphenylene sulfide (PPS) resin, a linear crystalline polymer, exhibits excellent melt fluidity due to the phenylene sulfide groups in its molecular backbone, making it a suitable material for melt-blown spinning [37]. In the melt-blown spinning process, the thermoplastic polymer is first sheared and melted by heating in a screw extruder. It is then quantitatively delivered into the melt-blown die by a metering pump. Inside the die, the melt is evenly distributed to the front spinneret plate. As the melt is extruded through the spinneret holes, high-speed, high-temperature airflow is ejected at a certain angle from both sides of the spinneret orifices. Under the action of this airflow, the melt is rapidly stretched and solidified into ultrafine fibers, which are deposited onto a collector. To enhance the mechanical strength of the ultrafine fibers, a pressing process is often applied under high temperature and pressure. The performance of the nonwoven separator is influenced by various factors, including the choice of polymer material, the setting of spinning parameters, and the parameters of the fiber-forming mold [75,76]. Melt-blown spinning offers high scalability and low production costs, and the resulting separators exhibit excellent thermal and mechanical stability. However, the process requires high operational precision, as fluctuations in the high-speed, high-temperature airflow may lead to particle dispersion within the fiber web, potentially affecting the performance of the lithium-ion battery separator.

#### 3.1.2. Electrospinning

The electrospinning methodology derives from investigations into electrically charged fluid dynamics, constituting a fabrication technique employed to synthesize polymeric fibers exhibiting diameters spanning from the micrometer to the nanometer scale under high-voltage electrostatic conditions [77,78]. Specifically, the spinning process includes the following steps: First, the polymer material is dissolved in a suitable solvent to form a homogeneous solution with a specific concentration and viscosity. Next, the solution is injected into a high-voltage electrostatic field using a metal needle. With progressive augmentation of the applied electric field intensity, Coulombic repulsion between surface-accumulated charges surpasses the solution’s cohesive interfacial energy, inducing Taylor cone formation at the capillary orifice and subsequent electrohydrodynamic extrusion of nanofibrous structures. The ejected nanofibers are stretched and entangled, randomly distributed on a collector to form a microporous nonwoven fabric, while the solvent evaporates during this process.

At room temperature, polyphenylene sulfide (PPS) is insoluble in any solvent, making the emerging solvent-free process of melt electrospinning a highly promising method for preparing PPS ultrafine fibers [79]. Melt electrospinning is similar to solution electrospinning, with the primary difference being that the polymer melt replaces the polymer solution. As a result, melt electrospinning imposes higher demands on equipment, requiring additional high-temperature heating devices to bring the polymer to a molten state. Moreover, polymer melts typically exhibit higher viscosity and lower conductivity, necessitating a stronger electric field to overcome the surface tension of the melt and stretch it into fibers. Although melt electrospinning currently has low production rates and is not yet scalable, it holds significant potential for applications where solvent handling or toxicity is a concern. The electrospinning process of polymeric materials is governed by a multitude of interdependent parameters, encompassing precursor solution/melt characteristics (rheological properties, electrical conductivity, interfacial energy), processing variables (applied electric field intensity, capillary orifice dimensions, collector displacement), and ambient conditions (thermal and hygrometric factors) [80,81,82]. Slight variations in these factors can significantly impact the overall morphology and performance of the fiber membrane, thereby affecting the performance of lithium-ion batteries [80,83,84]. Electrospinning is a simple, feasible, and effective technique for preparing fiber membranes with controllable nanostructures. However, its low production efficiency limits large-scale commercialization [85]. One intuitive solution to improve production efficiency is to increase the number of needles, but the repulsive electric fields generated by additional needles can cause deformation at the needle tips, leading to uneven nanofibers [86,87]. The rapidly developing needleless electrospinning technology offers an effective solution to this problem [88,89,90]. Compared to needle electrospinning, needleless electrospinning typically requires higher applied voltages and shorter collection distances to produce fibers within the same diameter range.

#### 3.1.3. Island-in-the-Sea Melt Spinning

Island-in-sea melt spinning is a method that uses two thermodynamically incompatible polymers as raw materials to produce bicomponent fibers with an island-in-sea structure through a specific spinning process. In this process, one polymer (referred to as the ‘island component’) is encapsulated by another polymer (the ‘sea component’), forming a unique island-in-sea structure. Ultrafine fibers are then prepared by chemically removing the ‘sea component’ using a specific solvent [91]. In the island-in-sea spinning process, the pre-crystallized polymers are first fed into their respective screw extruders, where they are heated, extruded, and melted. The molten polymers are then evenly distributed by the main and auxiliary metering pumps. In the spinning assembly, the ‘island component’ melt and the ‘sea component’ melt are mixed, and the blended melt is extruded through the spinneret. The extruded filaments are cooled by side-blown air and rapidly solidified into nascent fibers. Finally, depending on the linear density and type of fibers, different separation methods are applied. For example, xylene can be used to remove the polypropylene (PP) matrix phase from polyphenylene sulfide/polypropylene (PPS/PP) island-in-sea fibers to prepare PPS ultrafine fibers [92]. The fibers produced by island-in-sea spinning are extremely fine and have controllable morphology. However, the island-in-sea spinning process is complex and requires advanced spinning equipment. Additionally, the use of large amounts of volatile solvents to remove the ‘sea component’ poses challenges, as these solvents are often difficult to recycle. This not only increases the production cost of ultrafine fibers but also raises environmental pollution concerns.

### 3.2. Particle Leaching

Particle leaching is a commonly used, simple, and cost-effective technique for preparing porous materials. The specific steps of this process are as follows: Initially, the polymeric material undergoes dissolution in a compatible organic solvent medium, and then pore-forming templates of specific sizes (such as SiO_2_ nanoparticles or water-soluble salts) are incorporated into the solution. Subsequently, the homogeneous mixture is formed into a thin membrane via blade-coating or mold-casting methodologies. Following this, the solvent is eliminated through controlled evaporation or lyophilization. Ultimately, the templated polymeric membrane is subjected to immersion in a selective solvent system, and the dissolution of the templates results in the designed porous structure. Although particle leaching offers significant advantages in manufacturing highly porous separators, ensuring the interconnectivity of the resulting porous structures and improving the reproducibility of the preparation process remain challenges that need to be addressed [93,94].

### 3.3. Thermally Induced Phase Separation (TIPS)

Phase separation is a commonly used method for preparing lithium-ion battery separators. This method reduces internal stress and thermal shrinkage of pores, contributing to improved thermal stability of the separator [95]. Common phase separation techniques include non-solvent-induced phase separation (NIPS), thermally induced phase separation (TIPS), solvent evaporation-induced phase separation (SIPS), and vapor-induced phase separation (VIPS). Among these, thermally induced phase separation constitutes a robust methodology for preparing battery separators from polymers with poor solubility that cannot be processed using conventional methods. This technique primarily controls the membrane formation process by varying temperature. The preparation steps are as follows: First, the polymer material is dissolved in a good solvent at high temperatures to form a homogeneous polymer solution. Then, the polymer solution is coated or cast onto a flat substrate and solidified into a film. Subsequently, phase separation is induced through the evaporation of a volatile agent or by heating, resulting in the formation of polymer-rich and polymer-lean phases. Finally, the film is immersed in a diluent to remove the polymer-lean phase, and after complete solvent evaporation, a solidified microporous separator is obtained. The phase transition process in thermally induced phase separation is relatively simple, and the resulting porous separators exhibit high porosity, narrow pore size distribution, and excellent mechanical strength. However, this process is energy-intensive in practice and requires the consumption of large amounts of solvent.

### 3.4. Dry-Film Forming Process

The dry-film forming process is an innovative method for separator preparation that eliminates the use of solvents. It has garnered significant attention due to its advantages over the wet process, including simplicity, high production efficiency, minimal environmental pollution, and low cost. Research on the dry-film forming process for polyphenylene sulfide (PPS)-based separators dates back to 2013, when Zimmerman et al. successfully prepared PPS-based ion-doped solid-state separators using a traditional high-temperature polymer extrusion process. However, the high cost of this process hindered its application in large-scale production. Solvent-free dry film-forming technology is a core process in supercapacitor manufacturing and has broad applicability for producing various self-supporting films. In 2019, Zhou et al. applied this technology to the fabrication of PPS solid-state separators, successfully developing a highly crystalline, thin, and dense PPS solid-state separator [39]. The preparation process is as follows: First, crosslinked PPS is mixed with a small amount of tetrachloro-p-benzoquinone (TCBQ) and lithium chloride (LiCl) powder. The mixture undergoes hydrothermal reaction, distilled water washing, and drying to obtain pre-lithiated PPS powder. Next, the pre-lithiated PPS powder is thoroughly mixed with a small amount of polytetrafluoroethylene (PTFE). The mixture is subjected to supersonic airflow milling to fibrillate the PTFE, forming a network structure that immobilizes the PPS powder. Finally, a hot roller mill is used to perform multiple rolling processes at 130 °C to form a uniform and dense PPS solid-state separator. This method provides a practical pathway for the application of PPS separators, although the currently prepared separators still have some limitations, such as the need for improved mechanical strength.

## 4. Application of PPS Separators in Lithium Batteries

Currently, separators prepared from PPS resin are primarily used in lithium metal batteries and some higher energy density lithium metal-free batteries. Based on their pore structure, PPS separators can be broadly classified into two categories: porous separators and non-porous separators. This section will provide a detailed discussion on the characteristics, safety performance, and electrochemical performance of these two types of separators prepared using different processes. Table 1 summarizes the key parameters of various PPS separators, including thickness, porosity, electrolyte uptake, and ionic conductivity, offering a comprehensive reference for evaluating their performance.

Due to manufacturing process variations, polyphenylene sulfide (PPS) separators exhibit distinctly differentiated microstructural characteristics. From the perspective of porosity, they can be categorized into porous PPS separators fabricated via wet chemical processes and highly dense nonporous solid-state separators based on dry-film forming processes, as shown in Table 1. The former relies on electrolyte impregnated within pores to enable lithium-ion conduction, while the latter achieves ion transport through synergistic interaction between regular sulfur-site channels on highly crystalline PPS surfaces and solid–liquid biphasic lithium-ion conduction channels constructed by trace-amount infiltrated electrolyte. This architecture demonstrates enhanced solid-state ion transference numbers alongside homogenized electric field and ion flux characteristics.

### 4.1. Porous Separator

As mentioned earlier, a separator typically serves as both a physical barrier to prevent direct contact between the positive and negative electrodes and an efficient channel for lithium-ion transport. In PPS separators, a continuously connected pore structure can be formed through the random arrangement of fibers or achieved via processes such as particle leaching or phase inversion. To optimize separator performance, researchers have employed methods such as coating, grafting, and blending to regulate the pore structure, ensuring appropriate pore size and reasonable pore size distribution. This systematic optimization concurrently augments both the porosity and electrolyte absorption capacity of the separator while substantially enhancing the electrochemical performance of the battery system, consequently fulfilling the operational demands of high-energy-density energy storage devices.

#### 4.1.1. PPS/SiO_2_ Separator

**Figure 1:**
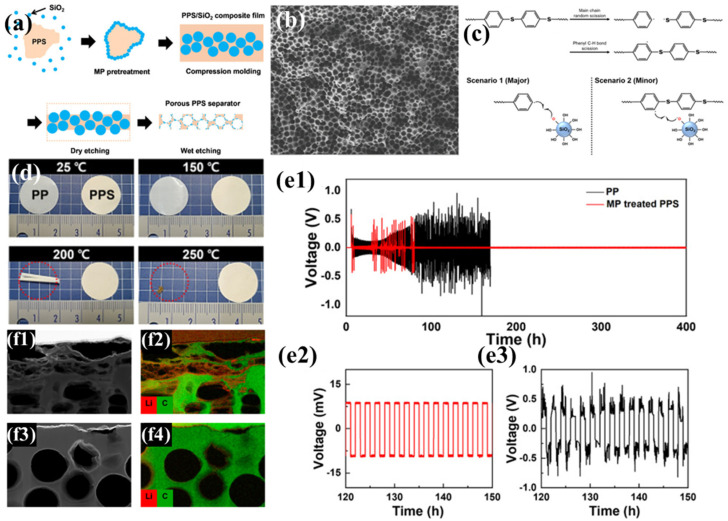
(**a**) Schematic representation of the fabrication process for porous polyphenylene sulfide (PPS) membranes. (**b**) PPS/SiO_2_ nanocomposite undergoing mechanochemical processing (MP) treatment. (**c**) Postulated mechanochemical reaction mechanism facilitating covalent bonding formation between PPS and SiO_2_ nanoparticles. (**d**) Thermomechanical deformation behavior of membrane specimens under incremental temperature variations. (**e**) Galvanostatic cycling performance of Li||Li symmetric cells over 400 h of operation, with insets (**e1**,**e2**) highlighting the 120–150 h interval at 1.0 mA/cm^2^ current density (**e3**), comparing MP-modified PPS separators with conventional polypropylene (PP) separators. (**f**) Transmission electron microscopy (TEM) micrographs (**f1**,**f3**) and corresponding electron energy-loss spectroscopy (EELS) elemental mapping for lithium and carbon distributions (**f2**,**f4**) in separators harvested after 400 h symmetric cell testing; (**f1**,**f2**) reference PP separator versus (**f3**,**f4**) MP-treated PPS separator. Reproduced with permission from reference [96]. Copyright 2021, Elsevier Ltd.

Kim et al. employed the particle leaching method, using spherical SiO_2_ nanoparticles as pore-forming agents and plasma-assisted mechanochemical processing (MP) to regulate the incompatible PPS/SiO_2_ interface, successfully achieving uniform distribution of SiO_2_ nanoparticles. Subsequently, the SiO_2_ phase was removed by etching, resulting in a PPS separator with a large number of uniformly distributed pores (Figure 1a) [96]. Compared to PPS separators prepared by melt-blown spinning and thermally induced phase separation, this method enables precise control of pore size (Figure 1b), avoiding issues such as excessively large pores and wide pore size distribution, thereby significantly improving electrolyte transport performance. During the MP process, mechanical forces cause uniform cleavage of C-S and C-H covalent bonds with lower dissociation energy in PPS, generating free radicals that serve as reactive sites, allowing SiO_2_ to chemically bond to PPS (Figure 1c). These bonded SiO_2_ particles are not removed during subsequent etching, and the polar functional groups remaining on the pore surfaces attract polar molecules, significantly enhancing the separator’s wettability with the electrolyte. Additionally, an MP-treated PPS separator exhibits no structural deformation below 250 °C, demonstrating excellent thermal stability (Figure 1d), indicating that it does not undergo severe shrinkage under thermal stress, which could otherwise lead to internal short circuits. In electrochemical performance tests (Figure 1e), an Li|PP|Li battery based on a traditional PP separator shows gradually increasing overpotential with significant fluctuations during cycling, and extremely high resistance after 100 h. In contrast, an Li|PPS|Li battery based on the MP-treated PPS separator exhibits stable overpotential with limited fluctuations over 400 h of cycling. This is attributed to severe damage caused by lithium dendrite blockage and pore collapse in the PP separator during cycling, whereas the MP-treated PPS separator effectively maintains the integrity of its pore structure (Figure 1f). Although slight lithium dendrite growth is observed in the PPS separator, it does not cause significant damage. Furthermore, the uniform mass transport pores in the PPS separator provide sites for uniform Li^+^ deposition, suppressing lithium dendrite accumulation on the electrode surface and further enhancing the battery’s cycling stability.

**Figure 2:**
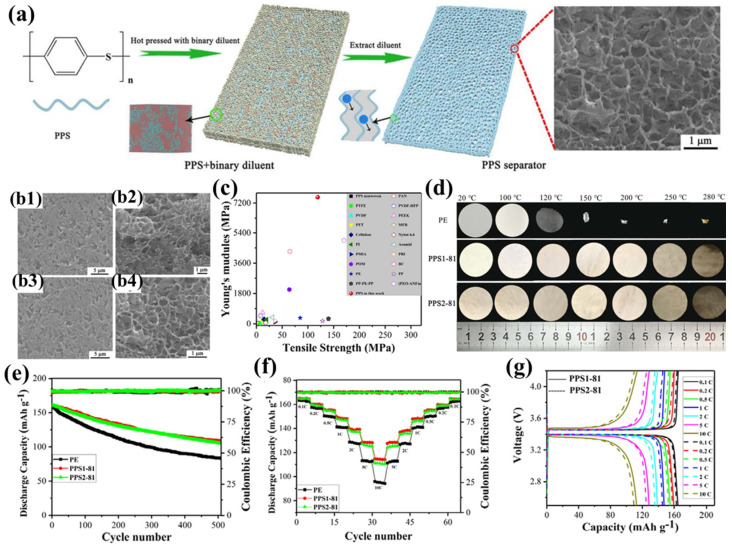
(**a**) Fabrication schematic of polyphenylene sulfide (PPS) separator membranes, with insets depicting mesoscale morphology DPD simulations and Li^+^ transport pathways. (**b**) Scanning electron micrographs of PPS1-81 and PPS2-81 separators: (**b1**,**b3**) surface topography; (**b2**,**b4**) cross-sectional architecture. (**c**) Mechanical property benchmarking: tensile strength and elastic modulus comparison between synthesized PPS separators and commercial polymeric counterparts (ambient conditions). (**d**) Thermomechanical stability assessment: dimensional evolution of PE, PPS1-81, and PPS2-81 separators under thermal stress (20–280 °C gradient). (**e**) Electrochemical cycling performance at 2C rate (voltage window: 2.5–4.2 V) comparing PE versus PPS-based separators. (**f**) Rate capability evaluation: discharge capacity retention across 0.1C-10C regimes. (**g**) Comparative analysis of PPS1-81 and PPS2-81 separator performance at varying C-rates. Reproduced with permission from reference [97]. Copyright 2018, Elsevier Ltd.

Liu et al. were the first to employ a binary diluent system to prepare PPS porous separators with a unique bicontinuous pore structure using thermally induced phase separation (TIPS) [106]. In their study [97], diphenyl sulfone (DPS) or benzophenone (DPK) was used as the first diluent, and polyethersulfone (PES) as the second diluent, to prepare the PPS1 and PPS2 series of separators, respectively (Figure 2a). Among them, the PPS1-81 and PPS2-81 separators exhibited the highest porosity and electrolyte uptake, primarily due to their unique tortuous three-dimensional interconnected pore structure, with an average pore diameter of approximately 500 nm and a narrow distribution (Figure 2b). This uniformly distributed submicron pore structure not only effectively suppresses lithium dendrite growth but also enables rapid and stable lithium-ion deposition, thereby mitigating self-discharge and preventing internal short circuits. Additionally, the polar groups in the separator form strong interactions with the carbonate electrolyte, further enhancing the separator’s wettability. The PPS separators demonstrated excellent tensile strength (119 MPa) and an extremely high Young’s modulus (7.55 GPa) (Figure 2c), enabling them to withstand the high tension during battery assembly and effectively address safety concerns arising from external mechanical wear, sudden temperature changes, or accidental high current densities. In particular, the ultra-high Young’s modulus significantly inhibits the growth of lithium dendrites toward the separator. Furthermore, the PPS separators maintained good mechanical integrity after being treated at temperatures ranging from 100 °C to 280 °C for 1 h, with a dimensional shrinkage rate of less than 2% even at 280 °C, demonstrating exceptional thermal stability (Figure 2d). Notably, the porous structure of the PPS separator remained intact at 250 °C, while the pore structure collapsed after 1 h of heat treatment at 280 °C, exhibiting a clear ‘shutdown’ function that effectively prevents internal short circuits and thermal runaway. Moreover, the high flame resistance and non-flammability of PPS resin further enhance the safety of the separator. Thanks to their unique pore structure and high porosity, the PPS1-81 and PPS2-81 separators provide abundant ion transport channels, while their excellent wettability and electrolyte uptake ensure sufficient lithium-ion supply. Additionally, the polar sulfur atoms in the separator promote lithium-ion migration, resulting in high lithium-ion conductivity and transference numbers. These characteristics enhance the interfacial adhesion at the separator/lithium metal anode interface, effectively mitigating charge transfer resistance during electrochemical cycling. Experiments showed that the Li|PPS|Li battery based on the PPS separator exhibited stable overpotential at a current density of 1 mA cm^−2^ (25 mV at 500 h and 30 mV at 2000 h), further confirming the advantages of the PPS separator in suppressing lithium dendrite growth, thereby enhancing the battery’s rate capability and cycling performance. Specifically, the half-cells based on the PPS1-81 and PPS2-81 separators retained capacities of 109.0 mAh g^−1^ and 105.7 mAh g^−1^, respectively, after 1500 cycles at a 2C rate, with capacity retention rates of 67.6% and 65.6%, respectively (Figure 2e). Furthermore, at current densities ranging from 0.1C to 10C, the discharge capacities of the PPS separators consistently exceeded those of commercial PE separators, and the capacity gap widened further as the current density increased (Figure 2f–g).

#### 4.1.2. PPS Composite Separator

Nonwoven membrane separators have attracted considerable research interest in lithium metal battery applications owing to their interconnected porous architecture that facilitates enhanced electrolyte permeation and improved ionic conductivity. However, pure PPS nonwoven separators, characterized by excessively large pore sizes and wide pore size distributions, struggle to effectively suppress lithium dendrite growth and self-discharge in lithium metal batteries, making them unsuitable for direct use as separators in such applications. To overcome this limitation, research on polyphenylene sulfide (PPS) fiber-based separators has primarily focused on developing PPS nonwoven-based composite separators. By incorporating other functional materials or coatings, composite structures with superior performance are constructed to meet the high-performance requirements of lithium metal batteries. These investigations yield critical mechanistic understanding for optimizing both the operational safety and electrochemical characteristics of lithium metal battery systems.

**Figure 3:**
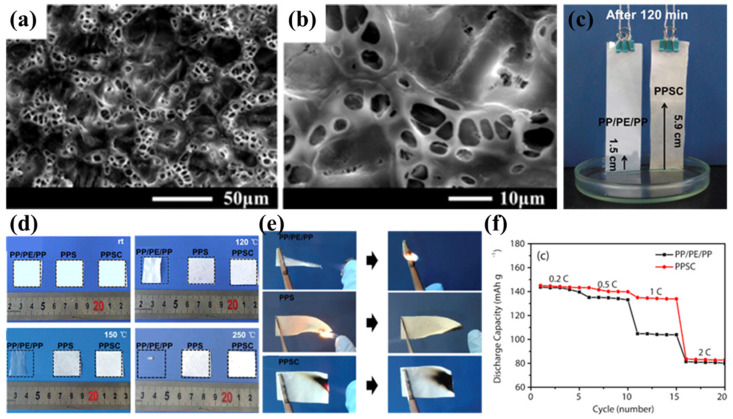
(**a**,**b**) Scanning electron micrographs of polyphenylene sulfide (PPS) nonwoven composite separators. (**c**) Comparative electrolyte wettability analysis: capillary rise height in commercial PP/PE/PP versus PPS/SiO_2_ composite (PPSC) separators after 120 min immersion. (**d**,**e**) Thermomechanical stability evaluation: dimensional integrity of conventional PP/PE/PP separator, pristine PPS nonwoven, and PPSC separator under isothermal conditions (120 °C, 150 °C, 250 °C for 30 min). (**f**) Rate capability comparison: electrochemical performance metrics of cells employing PP/PE/PP versus PPSC separators. Reproduced with permission from reference [98]. Copyright 2018, Elsevier Ltd.

Wang’s team successfully prepared PPS nonwoven fabric using the melt-blown spinning process [42]. However, owing to its excessive pore dimensions and non-uniform pore distribution, direct application as a lithium-ion battery separator resulted in undesirable phenomena including elevated self-discharge rates and heterogeneous current density profiles [107]. To address this limitation, Luo et al. proposed an improved solution by impregnating and coating the PPS nonwoven with the polymer polyvinylidene fluoride–hexafluoropropylene (PVDF-HFP) and inorganic nanoparticles (SiO_2_), successfully developing a composite separator based on nonwoven PPS (PPSC) [98]. During the preparation process, after solvent evaporation, the PPSC separator developed a highly advanced three-dimensional microstructure, exhibiting a uniformly distributed porous morphology (Figure 3a,b). This structure not only facilitates rapid absorption and retention of more electrolyte (Figure 3c) but also promotes lithium-ion transport between the electrodes, significantly enhancing lithium-ion conductivity. The PPSC separator maintained its original dimensions after heat treatment at temperatures ranging from 120 °C to 250 °C for 0.5 h, demonstrating excellent dimensional stability and flame-retardant properties (Figure 3d). This characteristic is primarily attributed to the high melting temperature of the PPS nonwoven substrate, which provides a robust framework for the PPSC separator. Additionally, the inorganic SiO_2_ nanoparticles in the coating further enhance the thermal resistance and flame-retardant properties of the composite separator, while the flame-retardant fluorine elements in PVDF-HFP also contribute synergistically (Figure 3e). At the same time, the highly polar coating material significantly improves the electrolyte wettability and uptake of the PPSC separator. The swelling behavior of PVDF-HFP in carbonate electrolytes not only increases electrolyte uptake but also enhances the compatibility between the separator and electrode interfaces, enabling the PPSC separator to consistently exhibit higher discharge capacities than commercial PP/PE/PP separators at current densities ranging from 0.2 C to 1 C (Figure 3f). However, due to the relatively large thickness of the separator, its discharge performance at higher current rates is somewhat limited. Although the PPSC battery demonstrates excellent initial capacity and Coulombic efficiency, its cycling performance is less stable, with rapid capacity decay, and even after 60 cycles, the capacity and Coulombic efficiency drop to relatively low levels. This phenomenon may be attributed to the excessively large pore size ultimately formed in the PPSC separator, which fails to effectively suppress lithium dendrite growth during long-term cycling. Additionally, the long-term swelling of PVDF-HFP in the electrolyte during cycling may cause it to detach from the substrate, potentially leading to micro-short circuits and further degradation of battery performance. This issue requires further research and resolution to enhance the long-term stability and practicality of the PPSC separator in lithium metal batteries.

**Figure 4:**
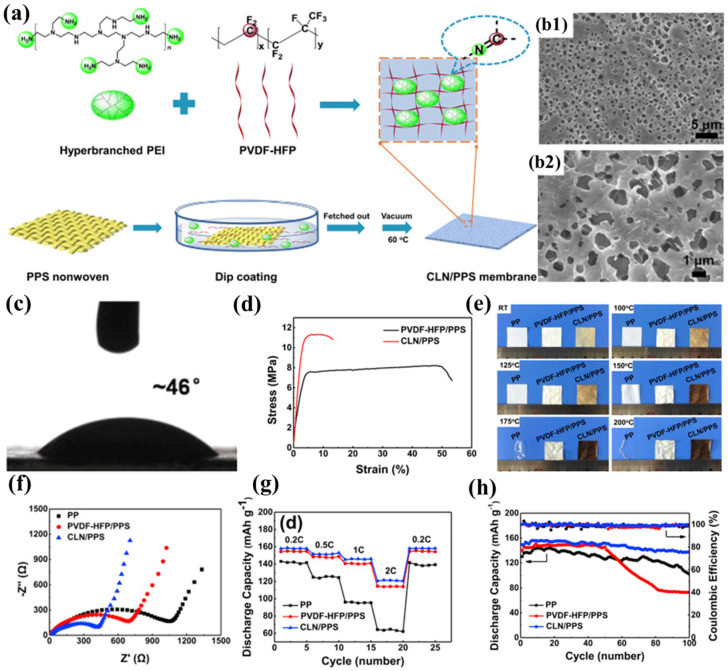
(**a**) Fabrication protocol for CLN/PPS composite separator and synthetic route for crosslinked polymeric electrolyte networks. (**b1**,**b2**) Scanning electron micrographs of CLN/PPS membrane morphology. (**c**) Electrolyte wettability analysis: contact angle measurements on CLN/PPS separator surface. (**d**) Mechanical properties: tensile stress–strain profiles of PVDF-HFP/PPS versus CLN/PPS membranes. (**e**) Thermomechanical stability assessment: dimensional integrity of commercial PP, PVDF-HFP/PPS, and CLN/PPS separators under isothermal annealing (100–200 °C, ΔT = 25 °C intervals, 30 min duration). (**f**) Electrochemical impedance spectroscopy: interfacial resistance characteristics of symmetric Li|separator|Li cells employing different separator materials. (**g**) Comparative rate performance evaluation: specific capacity versus C-rate for cells configured with various separators. (**h**) Long-term cycling stability: capacity retention profiles of cells utilizing different separator architectures. Reproduced with permission from reference [99]. Copyright 2020, Elsevier Ltd.

Zhang et al. successfully developed a novel composite separator (CLN/PPS) by constructing a cross-linked polymer electrolyte network on the surface of PPS nonwoven fabric through the chemical reaction between the highly polar C-F bonds in poly(vinylidene fluoride–hexafluoropropylene) (PVDF-HFP) and the nucleophilic amine groups in hyperbranched polyethyleneimine (PEI) (Figure 4a) [99]. The introduction of the polymer electrolyte effectively addressed the inherent defects of the nonwoven PPS, significantly reducing its pore size (0.5–2 μm) and creating a more uniform pore structure (Figure 4b1,b2). Additionally, the pore structure formed through solvent evaporation was denser and interconnected, providing ample storage space for the electrolyte, effectively suppressing lithium dendrite growth, and ensuring long-term stability of the embedded electrolyte. The stable network structure formed by the cross-linking reaction between PVDF-HFP and PEI made the CLN/PPS separator more compact, significantly increasing its Young’s modulus (439 MPa) and enhancing its resistance to deformation during battery assembly. Although its tensile strength (11.35 MPa) improved, it still lagged behind that of commercial separators (Figure 4d). Thanks to the heat-resistant skeleton provided by the PPS nonwoven substrate, the CLN/PPS separator exhibited negligible dimensional changes after being treated at 200 °C for 0.5 h, demonstrating excellent thermal stability (Figure 4e). Further cross-linking reactions at high temperatures caused the separator to darken in color. The highly polar nature and pronounced swelling characteristics of the polymer electrolyte substantially augmented the electrolyte retention capacity within the separator matrix, manifested through both immobilized and solvated phases. The amine functionalities in PEI improved interfacial compatibility with the electrolyte via hydrogen-bond interactions, while concurrently facilitating enhanced electrolyte uptake through molecular cavity encapsulation. Meanwhile, the presence of PEI disrupted the crystalline structure of PVDF-HFP, further increasing its swelling capacity for the electrolyte. The high electrolyte uptake endowed the CLN/PPS separator with high lithium-ion conductivity, ensuring sufficient lithium ions for battery operation. Additionally, the swelling behavior of the polymer electrolyte tightly immobilized the electrolyte, reducing side reactions and promoting close contact between the separator and electrodes, thereby improving interfacial compatibility and lowering interfacial impedance. Notably, the tertiary amine groups in PEI could consume acidic compounds (e.g., HF) generated in the electrolyte, preventing side reactions between the electrolyte and electrodes to some extent and further enhancing interfacial compatibility (Figure 4f). The superior ionic transport properties and optimal electrode-electrolyte interfacial characteristics synergistically contributed to the exceptional rate performance and cycling stability of the composite lithium nitride/polyphenylene sulfide (CLN/PPS) separator, demonstrating 91.8% capacity retention after 100 galvanostatic cycles at a 0.5C rate (Figure 4g), and its rate advantages became more pronounced as the current density increased (Figure 4h).

**Figure 5:**
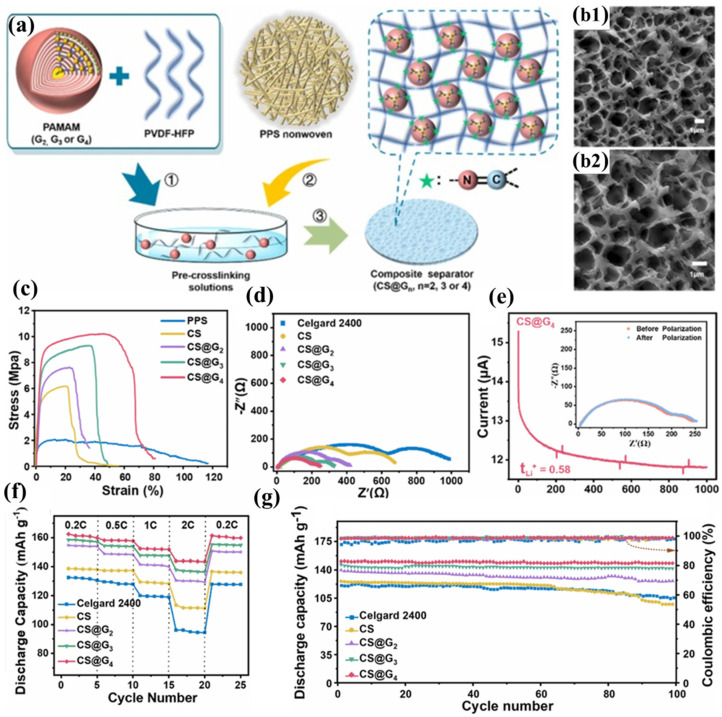
(**a**) Synthetic protocol for crosslinked polymeric coating deposition on polyphenylene sulfide (PPS) nonwoven substrate. (**b1**,**b2**) Scanning electron micrographs of CS@G4 composite membrane morphology. (**c**) Mechanical characterization: tensile stress–strain profiles of pristine PPS (CS), CS@G2,CS@G3, and CS@G4 membranes. (**d**) Electrochemical impedance spectroscopy: interfacial resistance characteristics of symmetric Li|separator|Li cells employing Celgard 2400 versus gradient crosslinked separators (CS series). (**e**) Lithium ion transference number analysis via chronoamperometry (applied potential: 10 mV; inset: Nyquist plots pre- and post-polarization). (**f**) Comparative rate capability assessment: specific capacity versus C-rate for cells configured with various separator architectures. (**g**) Long-term electrochemical cycling stability evaluation of cells incorporating different separator materials. Reproduced with permission from reference [100]. Copyright 2023, Elsevier Ltd.

Hu et al. successfully constructed a three-dimensional cross-linked network structure (CS@4) on the surface of PPS nonwoven fabric by replacing hyperbranched PEI with dendritic macromolecule polyamidoamine (PAMAM) to react with PVDF-HFP (Figure 5a) [100]. The multiple amine groups on the periphery of PAMAM can nucleophilically attack the C-F bonds in PVDF-HFP, forming a stable cross-linked structure (Figure 5b1,b2). Compared to PEI, PAMAM has a more complete and highly branched structure, enabling it to embed more electrolyte through its internal cavities. Furthermore, the spherical spatial structure of PAMAM increases the free volume of the polymer, further promoting electrolyte adsorption and lithium-ion transport. Additionally, the amide repeat units in PAMAM can interact with the electrolyte through hydrogen bonding. For these reasons, the CS@4 separator exhibits higher porosity, electrolyte wettability, electrolyte uptake, and lithium-ion conductivity than the CLN/PPS separator. Based on the same PPS nonwoven substrate and a similar cross-linked structure, the CS@4 separator demonstrates comparable mechanical properties (Figure 5c), thermal resistance, flame retardancy, and interfacial compatibility to the CLN/PPS separator (Figure 5d). The carbonyl and tertiary amine groups in PAMAM can adsorb more Li^+^, further promoting lithium-ion transport and achieving a lithium-ion transference number of 0.58 (Figure 5e). The high ionic conductivity, lithium-ion transference number, and excellent interfacial compatibility enable the CS@4 separator to exhibit superior rate performance at high current densities, with less polarization and higher discharge capacity (Figure 5f). The CS@4 separator tightly encapsulates the electrolyte through its narrow pore structure, the embedding effect of PAMAM’s internal cavities, and the hydrogen bonding interactions between PAMAM and the electrolyte, thereby maintaining a high electrolyte retention rate. The establishment of a three-dimensional crosslinked polymeric architecture reinforces the structural integrity of the coating layer, endowing it with exceptional resistance to prolonged electrolyte-induced swelling. This effectively prevents delamination of the functional coating while mitigating potential micro-short circuit formation within the cell. Consequently, batteries incorporating the CS@4 separator demonstrate remarkable cycling stability, maintaining 98.6% capacity retention after 100 galvanostatic charge–discharge cycles at a 0.5C rate (Figure 5g).

**Figure 6:**
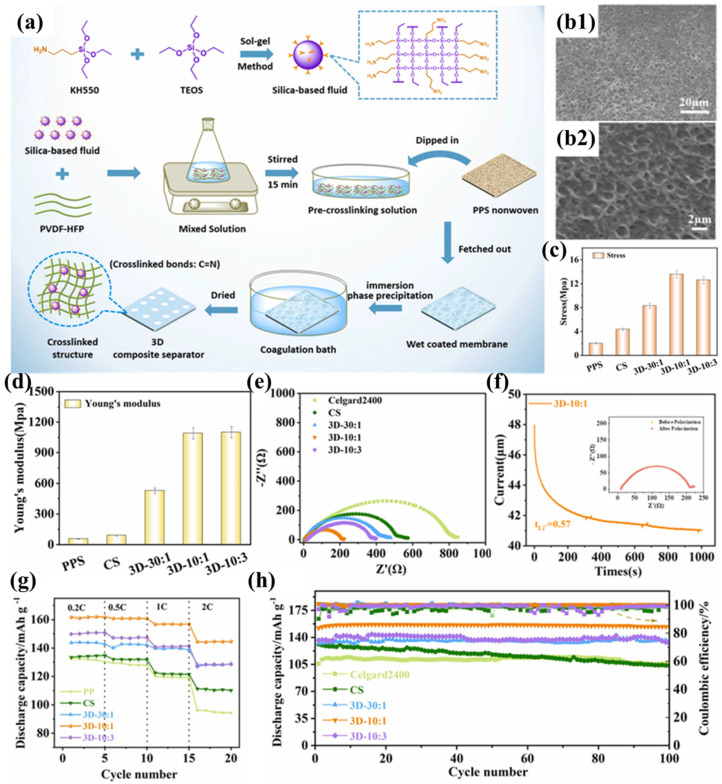
(**a**) Synthesis protocol for silica-based molecular fluid and coating deposition methodology for crosslinked architectures on PPS nonwoven substrates. (**b1**,**b2**) Scanning electron micrographs of 3D-10:1 separator morphology. (**c**) Mechanical strength characterization: tensile resistance measurements. (**d**) Elastic modulus determination: Young’s modulus evaluation. (**e**) Electrochemical impedance spectroscopy: interfacial resistance profiles in Li||separator||Li symmetric cells. (**f**) Lithium ion transference number analysis via chronoamperometry (applied potential: 10 mV; inset: comparative Nyquist plots pre- and post-polarization). (**g**) Comparative rate capability assessment: specific capacity versus C-rate for cells with various separators. (**h**) Long-term cycling stability evaluation: capacity retention versus cycle number. Reproduced with permission from reference [101]. Copyright 2024, Elsevier Ltd.

Zhu et al. successfully prepared a cross-linked composite separator (3D-10:1) by impregnating and coating PPS nonwoven fabric with silica-based nanofluids and PVDF-HFP (Figure 6a) [101]. Compared to traditional nanoparticles, silica-based nanofluids exhibit liquid-like properties and are less prone to aggregation in the polymer matrix. More importantly, by introducing terminal amine groups into the nanofluid molecules, they can react with PVDF-HFP to form a three-dimensional cross-linked network structure coating. Simultaneously, a sponge-like pore structure was formed in the coating using the immersion phase inversion method (Figure 6b1,b2), where the pore size formed by solvent diffusion into the coagulation bath is influenced by the degree of polymer entanglement. With increasing incorporation of silicon-based fluid, the crosslinking density within the system exhibits progressive enhancement, resulting in amplified interchain entanglement of polymeric networks and the development of diminished pore architectures. When the ratio of PVDF-HFP to fluid is 10:1, the pore size reaches its minimum, making it more suitable as a battery separator to suppress micro-short circuits. Nevertheless, the membrane with a 3D-10:3 composition ratio displayed anomalous formation of macroporous structures. Beyond a critical concentration threshold, bond saturation phenomena impede additional crosslinking participation of the fluid molecules to augment polymer chain interactions. Furthermore, superfluous fluid introduction modifies the phase separation kinetics during liquid–liquid exchange, consequently diminishing the composite’s total void fraction. The silica-based fluid enhances the affinity between the separator and the electrolyte through polar and hydrogen bonding interactions, facilitating electrolyte capture and increasing the fixed-phase electrolyte within the separator. The profuse interconnected pore structure of the 3D-10:1 separator promotes electrolyte infiltration and diffusion, while the free-phase electrolyte in the pore structure further enhances electrolyte storage capacity, resulting in excellent electrolyte wettability and uptake. Compared to the PPSC separator modified with traditional SiO_2_ nanoparticles, the 3D-10:1 separator exhibits equally excellent thermal resistance and flame retardancy, but its three-dimensional cross-linked network structure significantly improves tensile strength (13.58 MPa) (Figure 6c) and Young’s modulus (Figure 6d). The good electrolyte wettability and uptake ensure high ionic conductivity for the 3D-10:1 separator and help reduce intrinsic and interfacial impedance. Furthermore, the polymeric constituent effectively reduces the interfacial charge transfer resistance at the electrode-separator interface, thereby improving interfacial adhesion characteristics (Figure 6e). The introduction of silica-based fluids enables the composite separator to attract anions through terminal hydroxyl groups (-OH), promoting the full dissociation of Li^+^ and accelerating Li^+^ percolation and migration (Figure 6f), thereby significantly reducing the concentration gradient in the battery and mitigating polarization under high current densities. This results in outstanding rate performance for the 3D-10:1 separator (Figure 6g). The electrochemical cell employing the 3D-10:1 separator exhibits remarkable cycling durability, maintaining 98.5% capacity retention following 100 galvanostatic cycles at a 0.5C discharge rate (Figure 6h).

**Figure 7:**
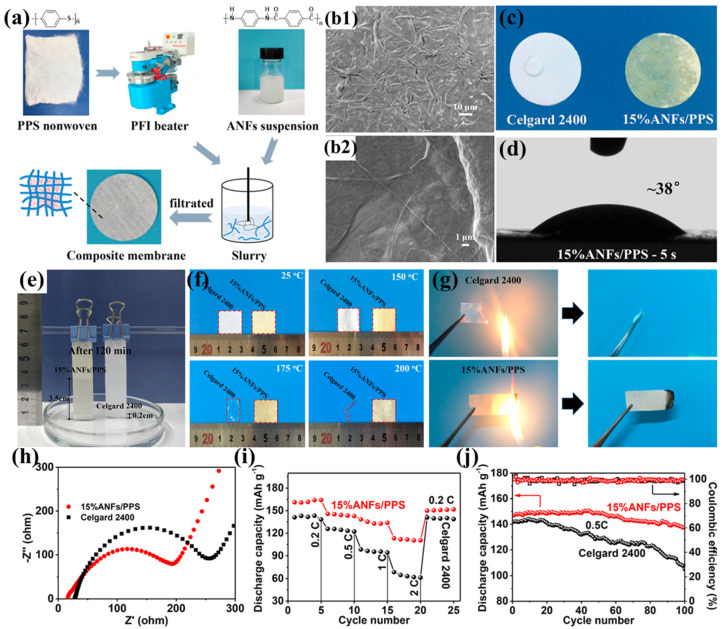
(**a**) Fabrication schematic of aramid nanofiber/polyphenylene sulfide (ANF/PPS) composite membranes. (**b1**,**b2**) Scanning electron micrographs of 15% ANFs/PPS composite membrane at varying magnifications. (**c**) Electrolyte wettability assessment: surface absorption dynamics 30 s post deposition. (**d**) Contact angle measurements of ANF/PPS separator (2 μL electrolyte deposition, 5 s equilibrium). (**e**) Capillary rise comparison: electrolyte infiltration height after 120 min (Celgard 2400 vs. 15% ANF/PPS). (**f**) Thermomechanical stability: dimensional integrity before/after isothermal treatment (RT, 150 °C, 175 °C, 200 °C; 30 min duration). (**g**) Flammability resistance evaluation: combustion behavior of Celgard 2400 versus ANF/PPS separators. (**h**) Electrochemical impedance spectroscopy: interfacial resistance in Li||separator||Li symmetric cells. (**i**) Rate capability analysis: discharge capacity versus C-rate for cells with different separators. (**j**) Long-term cycling performance at 0.5C charge/discharge current density. Reproduced with permission from reference [102]. Copyright 2024, Elsevier Ltd.

Zhu et al. prepared a highly resistant aramid nanofiber/polyphenylene sulfide nonwoven composite separator (ANF/PPS) using a simple papermaking method with PPS nonwoven and aramid nanofibers (ANFs) as raw materials (Figure 7a) [102]. When the ANF content increased to 15%, the ANFs completely covered and tightly wrapped the PPS fibers, enabling the ANF/PPS separator to form nanoscale pores and a three-dimensional porous structure (Figure 7b1,b2). This structure facilitates rapid absorption and immobilization of more electrolyte, thereby promoting lithium-ion transport, enhancing lithium-ion conductivity, and significantly improving electrochemical performance. Additionally, the hydrophilic amide polar groups in ANFs promote the absorption and diffusion of the electrolyte within the separator, further enhancing the wettability of the ANF/PPS separator (Figure 7c–e). The tightly woven network formed by ANFs significantly improves the tensile strength of the separator (9.8 MPa). Although it still lags behind commercial separators, its high Young’s modulus (528 MPa) effectively maintains mechanical integrity, preventing separator rupture due to accidental impacts, while also helping to suppress lithium dendrite growth during battery cycling. The superior thermal resistance exhibited by both the PPS fibrous matrix and aramid ANFs collectively guarantees the thermostability of the ANFs/PPS composite separator (Figure 7f), while the high limiting oxygen index of PPS (46) and poly(p-phenylene terephthalamide) (PPTA, 29) endows the separator with excellent flame-retardant properties (Figure 7g), making it sufficiently safe for high-power lithium-ion battery applications. Thanks to its high ionic conductivity and good interfacial compatibility with electrodes (Figure 7h), the battery based on the ANF/PPS separator exhibits excellent rate performance. Within the current density range of 0.2C to 2C, its discharge capacity consistently exceeds that of the battery based on the Celgard 2400 separator (Figure 7i). Furthermore, the ANFs/PPS separator achieves a capacity retention rate of 92% after 100 charge–discharge cycles at 0.5C, significantly outperforming the 73% retention rate of the Celgard 2400 separator (Figure 7j). This excellent cycling performance is primarily attributed to the increased Young’s modulus provided by ANFs and the three-dimensional microporous network structure they construct, which effectively suppress lithium dendrite growth and prevent short circuits during long-term cycling.

**Figure 8:**
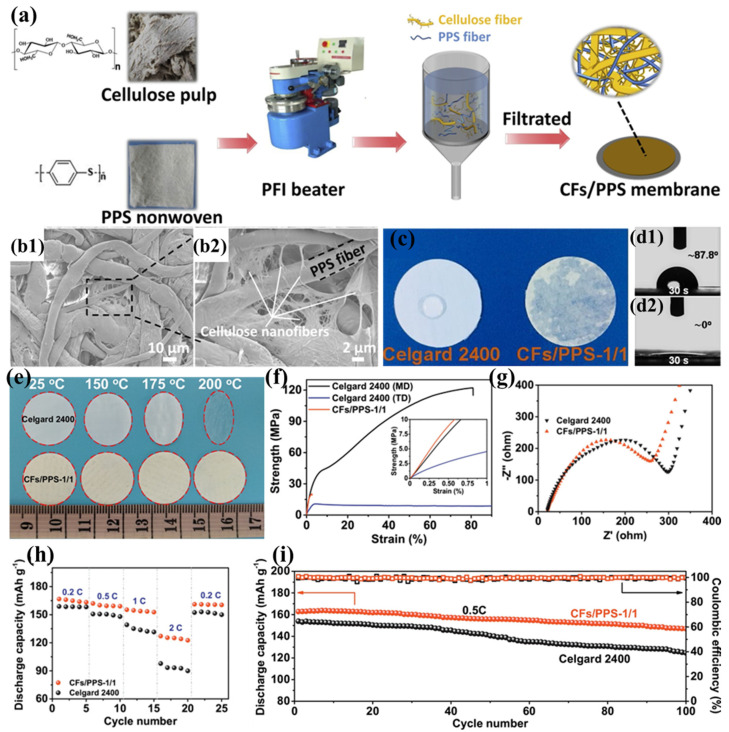
(**a**) Fabrication schematic of carbon fiber/polyphenylene sulfide (CFs/PPS) composite membranes. (**b1**,**b2**) Representative scanning electron micrographs of CF/PPS-1:1 composite membrane morphology. (**c**) Electrolyte wettability analysis: surface diffusion dynamics on membrane substrates. (**d1**,**d2**) Comparative contact angle measurements (30 s equilibrium) for Celgard 2400 versus CF/PPS-1:1 membranes. (**e**) Dimensional stability assessment: thermal shrinkage behavior after isothermal treatment (RT, 150 °C, 175 °C, 200 °C; 30 min duration). (**f**) Mechanical property evaluation: tensile stress–strain profiles in machine direction (MD) and transverse direction (TD) (inset: 0–1% strain region magnification). (**g**) Electrochemical impedance spectroscopy: interfacial characteristics of Li||separator||Li symmetric cells. (**h**) Rate capability comparison: discharge capacity versus C-rate for cells employing different separator architectures. (**i**) Cyclic performance analysis: capacity retention over 100 cycles at 0.5C charge/discharge rates. Reproduced with permission from reference [103]. Copyright 2020, Elsevier Ltd.

The substantial production costs associated with PPTA nanofibers present a significant barrier to their widespread implementation in lithium-ion battery separator technologies. To mitigate this challenge, Zhu et al. proposed a cost-effective alternative by blending cellulose fibers (CFs) with PPS fibers to prepare a novel composite separator (CF/PPS) (Figure 8a) [103]. During the pulping process, cellulose fibers are broken down into numerous nanofibers (approximately 300 nm), which interweave with PPS fibers to create curved voids (Figure 8b1,b2), effectively compensating for the excessively large pores of pure PPS fibers. As the CF content increases, more and smaller pore structures appear in the composite separator. When the ratio of CFs to PPS is 1:1, the fiber pores are minimized and exhibit a narrow distribution, making it an ideal separator material. This three-dimensional porous architecture significantly improves electrolyte retention while simultaneously inhibiting lithium dendrite formation. Furthermore, the abundant hydroxyl groups in cellulose immobilize the electrolyte through hydrogen bonding, further improving the separator’s conductivity. Thanks to the inherent lyophilicity of cellulose and the well-interpenetrated porous structure of the separator, the CF/PPS separator exhibits excellent electrolyte wettability (Figure 8c,d2). The thermal stability of both cellulose and PPS fibers ensures the superior heat resistance of the CF/PPS separator (Figure 8e). Meanwhile, the interwoven fiber structure and the rich hydrogen bonding between cellulose fibers endow the separator with good mechanical properties (tensile strength of 20.52 MPa) (Figure 8f). The excellent ionic conductivity and good interfacial compatibility of the CF/PPS separator (Figure 8g) significantly mitigate ohmic polarization and enhance cycling stability. The battery based on the CF/PPS separator achieves a discharge capacity of 127.1 mAh g^−1^ at a 2C rate (Figure 8h) and a capacity retention rate of 90.3% after 100 charge–discharge cycles at 0.5C (Figure 8i).

**Figure 9:**
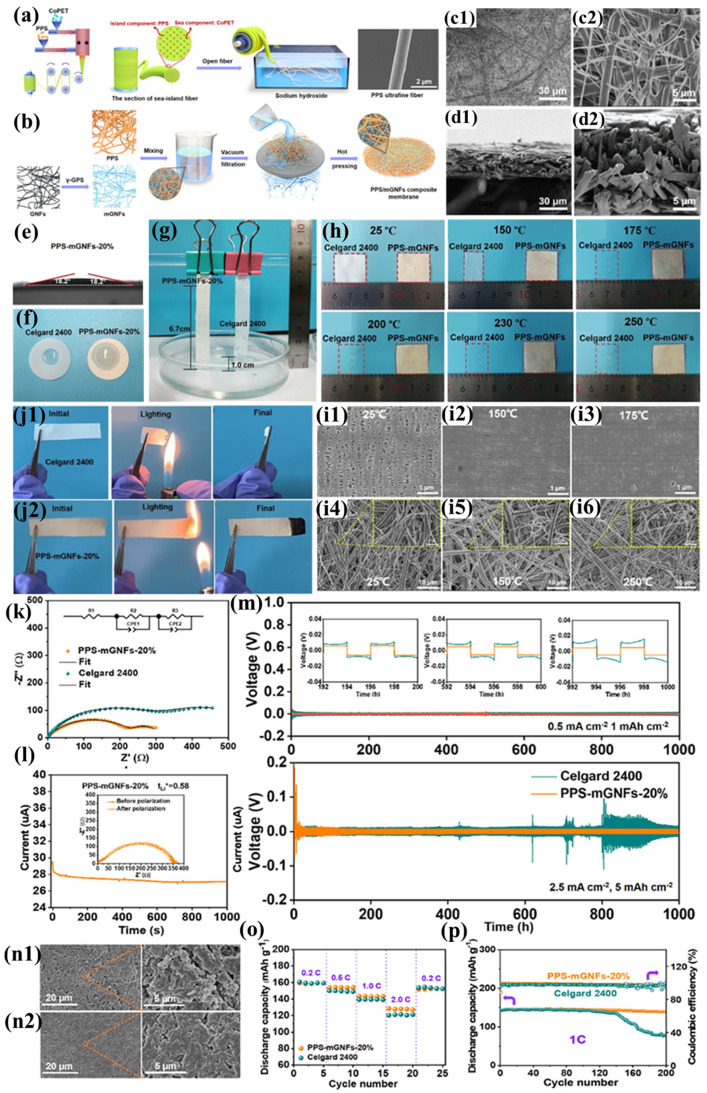
(**a**) Synthetic protocol for PPS sea-island micro/nanofiber fabrication. (**b**) Manufacturing schematic of PPS-mGNF membranes. (**c1**,**c2**) Surface morphology characterization of PPS-mGNFs via scanning electron microscopy. (**d1**,**d2**) Cross-sectional microstructure analysis of PPS-mGNF membranes. (**e**) Electrolyte wettability assessment: static contact angle measurements. (**f**) Electrolyte droplet spreading dynamics on membrane surfaces. (**g**) Quantitative electrolyte absorption capacity after 2 h immersion. (**h**) Comparative thermomechanical stability: dimensional evolution of Celgard 2400 versus PPS-mGNFs-20% under thermal stress (25–250 °C, 30 min isothermal). (**i1**–**i3**) Temperature-dependent morphological evolution of Celgard 2400. (**i4**–**i6**) Thermal stability evaluation of PPS-mGNF microstructure. (**j1**,**j2**) Flammability resistance comparison: combustion behavior analysis. (**k**) Electrochemical impedance spectroscopy: interfacial resistance characteristics. (**l**) Lithium ion transference number determination via chronoamperometry (10 mV bias; inset: pre-/post-polarization Nyquist plots). (**m**) Galvanostatic cycling profiles of symmetric cells under varying current densities (0.5–2.5 mA cm^−2^). (**n1**,**n2**) Post-cycling electrode morphology characterization after 1000 h operation. (**o**) Comparative rate capability assessment at multiple C-rates. (**p**) Long-term electrochemical cycling stability evaluation. Reproduced with permission from reference [104]. Copyright 2023, Elsevier Ltd.

Yu et al. prepared PPS ultrafine fiber nonwoven fabric using the island-in-sea spinning process. Compared to the PPS fiber nonwoven fabric prepared by Wang’s team using melt-blown spinning, the resulting fabric exhibited smaller pores (0.8–1 μm), finer and more uniformly distributed fiber diameters, thinner thickness, and superior mechanical properties (Figure 9a). By employing a simple papermaking method, uniform PPS ultrafine fibers were blended with glass nanofibers (GNFs) to successfully prepare a polyphenylene sulfide/glass fiber (PPS-mGNF) composite separator with controllable porosity and pore size (Figure 9b) [104]. This separator was not only formed through a hot-pressing process but also modified using a silane coupling agent (γ-glycidoxypropyltrimethoxysilane, γ-GPS). One end of γ-GPS connects to the glass fibers via hydrogen bonding, while the other end reacts with hydroxyl groups on PPS through epoxy groups, forming a dense hierarchical cross-linked structure (Figure 9c1,d2). This modification substantially improves the mechanical robustness of the separator, exhibiting enhanced flexibility coupled with superior tensile characteristics (demonstrating a tensile strength of 22.2 MPa and elastic modulus of 628.9 MPa). This hierarchical cross-linked structure not only imparts high porosity to the separator but also combines the strong polarity of mGNFs, resulting in excellent electrolyte wettability and uptake (Figure 9e–g), thereby promoting Li^+^ transport and enhancing ionic conductivity. Additionally, the high tension and flexibility of the separator effectively reduce the risk of damage caused by accidental collisions during battery assembly and prevent internal short circuits and lithium dendrite growth due to rough electrode fragments. The PPS-mGNF separator also demonstrates exceptional thermal stability (Figure 8h), preserving a well-defined porous architecture following thermal exposure at 250 °C for 30 min (Figure 9i). The radicals generated during the combustion of PPS react with bicyclic carbon atoms to form a dense carbonized layer, preventing the transfer of heat and oxygen, thereby endowing the separator with excellent flame retardancy (Figure 9j1,j2). Meanwhile, the introduced non-flammable inorganic GNFs further hinder flame propagation, significantly improving the flammability resistance characteristics of the separator membrane. Thanks to the separator’s good electrolyte wettability and absorption capacity, both its bulk resistance and interfacial resistance are low (Figure 9k), effectively mitigating ohmic polarization. Furthermore, the cross-linked network of the separator restricts anion mobility through physical binding with PF_6_^-^ ions, promoting Li^+^ transport. Simultaneously, the Si-O units in γ-GPS capture electrolyte anions, making the separator surface a Lewis acid center and further increasing the available Li^+^ for conduction (Figure 9l), thereby reducing concentration polarization. These characteristics endow the PPS-mGNF separator with exceptional rate capability, outperforming conventional Celgard 2400 separators under high-current-density operating conditions (Figure 9o). Electrochemical cells employing conventional Celgard 2400 separators exhibit significant capacity degradation (>40%) following 140 galvanostatic cycles at 1C rate. In contrast, cells incorporating PPS-mGNF separators retain stable discharge capacities of 140 mAh g^−1^ after 200 cycles, demonstrating superior long-term cycling performance (Figure 9p). The enhanced performance stems from the three-dimensional interconnected network within the PPS-mGNF membrane, offering exceptional electrolyte wettability and numerous Li^+^ adsorption centers, thus optimizing the battery’s cycling stability. The Li|Celgard 2400|Li battery exhibits a high overpotential of 15 mV at a current density of 0.5 mA cm^−2^, with significant voltage fluctuations, while the Li|PPS-mGNF|Li battery shows an overpotential of only 8.7 mV at 2.5 mA cm^−2^, with stable voltage evolution and minimal hysteresis during cycling, demonstrating excellent electrochemical performance and a stable voltage platform (Figure 9m). Such voltage fluctuations are often attributed to lithium dendrite growth during lithium-ion stripping and deposition. Additionally, the surface of the Li|Celgard 2400|Li battery’s negative electrode becomes rough after cycling (Figure 9n2), whereas the surface of the Li|PPS-mGNF|Li battery’s negative electrode remains relatively smooth and dense, with no obvious lithium dendrite structures (Figure 9n2). This indicates that the PPS-mGNF separator can establish a low-polarization and stable interface, effectively suppressing lithium dendrite growth. This is the result of the synergistic effect of the PPS-mGNF separator’s high Young’s modulus and the stable SEI layer formed during cycling. The novel separator prepared by blending two fibers using the papermaking method achieves significant improvements in mechanical properties, cycling performance, and rate performance. However, due to the insufficient structural stability of the physically cross-linked woven structure, the separator may have a shorter lifespan, making it difficult to meet long-term usage requirements.

Through strategic pore structure engineering of PPS nonwoven matrices via nanoparticle incorporation, investigators have markedly improved the mechanical robustness of PPS separators while simultaneously optimizing battery electrochemical characteristics, constructing polymer electrolyte coatings, and blending with nanofibers. These modifications have resulted in higher porosity and electrolyte uptake. The strategic incorporation of polar functional groups substantially enhances the electrolyte-philic properties and interfacial wettability of PPS nonwoven matrices, a critical determinant for optimizing separator electrochemical functionality. However, PPS nonwoven-based composite separators generally suffer from excessive thickness. This design of thick separators occupies space within the battery that could otherwise be used for active materials, consequently constraining the advancement of next-generation high-energy-density lithium-ion battery technologies [24]. Additionally, the complex preparation processes and high manufacturing costs further hinder their industrialization.

### 4.2. Non-Porous Separator

Lithium metal batteries and lithium metal-free battery systems have attracted considerable research interest owing to their exceptional energy density characteristics. Nevertheless, the inhomogeneous Li^+^ electrodeposition at the anode interface and subsequent dendritic lithium propagation through separator pore channels, resulting in internal cell short-circuiting, present substantial obstacles to their practical implementation. To address this issue, researchers have proposed the concept of non-porous separators [108]. The primary advantage of such separators lies in their ability to effectively suppress the growth of lithium dendrites on the anode, thereby preventing internal short circuits. This innovation provides new research directions and technical insights for developing PPS-based separators suitable for lithium metal batteries and lithium metal-free batteries.

Zhou et al. prepared a highly crystalline non-porous polyphenylene sulfide solid-state separator (PPS-SSS) using a dry-film forming process [39]. Unlike traditional separators, the PPS-SSS separator features a unique Li^+^ transport mechanism that does not rely entirely on pore structures for Li^+^ transmission. Specifically, Li^+^ transport in the PPS-SSS separator follows three fixed pathways: first, along the sulfur atom channels in PPS crystals; second, along the molecular chains on the surface of PPS particles; and third, through the liquid-phase infiltrated gaps in the separator. This diversified Li^+^ transport pathway, combined with a unique anion immobilization mechanism, endows the separator with high ionic conductivity and ion transference numbers. Additionally, the diffusion of Li^+^ along fixed pathways ensures uniform Li^+^ flux, promoting uniform Li^+^ deposition on the anode surface and effectively suppressing lithium dendrite growth. However, the PPS-SSS separator exhibits extremely low tensile strength and cannot withstand the tension generated during battery assembly. Furthermore, orbital calculations suggest that electron delocalization in the carbon chain may confer conductivity to PTFE. Therefore, a PE separator was coated on the anode side of the PPS-SSS separator to create a PE-PPS composite solid-state separator (PE-PPS-CSSS). The unique advantage of the PE-PPS-CSSS separator lies in its ability to maintain a high ion transport rate under minimal electrolyte conditions while effectively suppressing lithium dendrite growth at high current densities. Additionally, its high thermal stability and the flame-retardant properties of PPS further enhance battery safety. Nevertheless, the highly crystalline PPS powder exhibits considerable rigidity, yielding a separator membrane with inferior mechanical strength, limited fracture resistance, and substantial surface porosity. These characteristics not only hinder battery assembly but may also negatively impact electrochemical performance.

**Figure 10:**
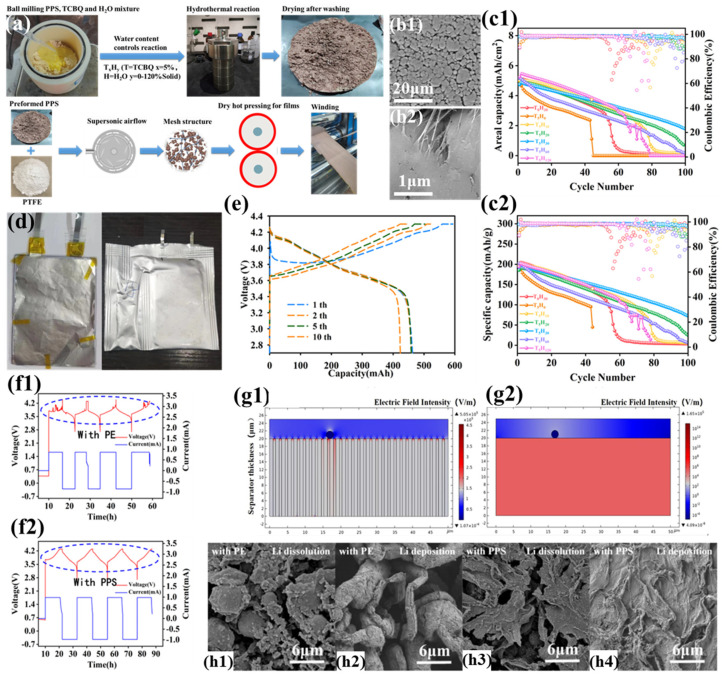
(**a**) Manufacturing process schematic of PPS solid-state separator (T_5_H_30_). (**b1**,**b2**) Surface morphology characterization of PPS-SSS (T_5_H_30_) via scanning electron microscopy. (**c1**,**c2**) Electrochemical cycling performance of lithium metal-free configurations employing seven PPS-SSS variants at 0.1C rate (20 mA g^−1^, ∼0.5 mA cm^−2^). (**d**) Structural configuration and exterior morphology of flexible-packaged lithium metal-free battery. (**e**) Galvanostatic charge–discharge profiles of flexible lithium metal-free battery. (**f1**,**f2**) Voltage-current characteristics during cycling of CuSn anode cells contaminated with 15 μm Cu particulates, comparing Al_2_O_3_-coated PE separators with PPS-SSS(T_5_H_30_). (**g**) Finite element modeling (COMSOL) of electric field distribution contrasts induced by Cu contaminants in porous PE versus dense PPS-SSS(T_5_H_30_) separators. (**h1**,**h2**) Post-cycling SEM analysis of CuSn anodes (15 μm Cu contamination) with Al_2_O_3_-PE separators under Li dissolution/deposition conditions. (**h3**,**h4**) Corresponding anode morphology evaluation for PPS-SSS(T_5_H_30_) configurations. Reproduced with permission from reference [105]. Copyright 2023, Elsevier Ltd.

To address the aforementioned issues, Zhou et al. introduced a chlorocatechol crosslinker onto the surface of highly crystalline PPS powder using a hydrothermal process, successfully improving the performance of the PPS-SSS separator (Figure 10a) [105]. The introduction of the crosslinker resulted in a smoother surface and a denser, pore-free structure for the separator (Figure 10b1,b2), significantly enhancing its mechanical properties (tensile strength of 4 MPa). This pore-free structure enables more uniform ion flux, reduces lithium dendrite deposition, and suppresses the consumption of active lithium and electrolyte during cycling. Additionally, the pore-free structure avoids self-discharge and lithium dendrite growth caused by localized breakdown under high discharge rates or extreme conditions. Despite the lack of traditional pore-based lithium-ion transport channels, the separator still exhibits high ionic conductivity and lithium-ion transference numbers (0.8). Thanks to PPS’s melting point of nearly 300 °C, the pore-free PPS-based solid-state separator demonstrates excellent thermal stability. When subjected to regional temperature excursions in the electrochemical cell, the separator provides additional reaction time for the electronic control system, effectively preventing safety incidents. The NCM811||Li cell incorporating this advanced separator demonstrates superior electrochemical performance, delivering a specific discharge capacity of 226 mAh g^−1^ with remarkable cycling stability at ambient temperature. Significantly, a lithium metal-free configuration utilizing the PPS-SSS separator, paired with a 10 μm tin-plated copper foil anode (CuSn) and NCM811 cathode, achieves exceptional areal capacity (5 mAh cm^−2^) and specific capacity (200 mAh g^−1^), as illustrated in Figure 10c1,c2. This configuration attains an impressive energy density surpassing 440 Wh kg^−1^ (Figure 10d,e) (discharge state: 1700 WhL^−1^, charge state: 1100 WhL^−1^). Under standard assembly conditions, the electrochemical cell maintains 80% capacity retention through 40 charge–discharge cycles, with progressive degradation to 40% retention after 100 cycles. The most prominent advantage of the PPS-SSS separator lies in its ability to effectively suppress lithium dendrite growth on the anode. Through deliberate deposition of copper particulate contaminants onto the anode interface, this study replicates the deleterious effects of metallic impurities potentially generated during large-scale battery manufacturing processes on electrochemical performance. The battery using a commercial PE separator exhibited a jagged charging curve and failed to achieve constant-voltage charging at 4.3 V (Figure 10f1), while the battery using the PPS solid-state separator displayed normal charge–discharge curves and stable current output (Figure 10f2). Multiphysics simulations analyzing copper particulate effects on separator field distributions demonstrated that metallic contaminants induce localized electric field intensification in adjacent polyethylene separator pores, resulting in heterogeneous lithium-ion flux profiles (Figure 10g1) that precipitate dendritic lithium formation. In contrast, the dense PPS separator maintains a uniform internal electric field distribution, unaffected by Cu powder particles (Figure 10g2), ensuring battery stability during charge–discharge cycles. In a lithium metal-free battery using a commercial PE separator, needle-like residual lithium dendrites were deposited on the Cu powder surface under discharge/Li dissolution conditions (Figure 10h1), while during charging/lithium deposition, the lithium dendrites thickened and elongated further (Figure 10h2), amplifying the heterogeneity of electric field distribution across the separator pore architecture. In stark contrast, in the PPS solid-state separator-based lithium metal-free battery, lithium metal was uniformly deposited in a sheet-like form (Figure 10h3). During discharge/dissolution, residual plate-like lithium deposits covered the Cu powder particles on the anode surface (Figure 10h4). This observed behavior originates from the electric field and ion flux homogenization capability of the PPS-based solid-state separator, coupled with the synergistic stabilization provided by the SEI layer during cycling.

The non-porous PPS-SSS exhibits exceptional rate capability and cycling durability in high-energy-density battery systems, attributable to its distinctive ion conduction pathway. Additionally, even when metal particle contaminants are introduced to the anode, this separator architecture demonstrates significant efficacy in inhibiting lithium dendritic propagation, consequently mitigating the occurrence of internal cell short-circuiting. However, its preparation process relies on fibrillated PTFE to form a spatial network structure that immobilizes PPS particles, and the oxidized PTFE exhibits conductivity. This necessitates the combination of the PPS-SSS separator with a PE separator to enhance insulation. Unfortunately, the inherent drawbacks of the PE separator may, to some extent, compromise the overall safety of the battery.

Additionally, the PPS-SSS separator demonstrates strong compatibility, excelling not only in lithium metal batteries but also showing promising potential in sodium-ion batteries [109] and aqueous zinc-ion batteries [106].

A sodium-ion battery study [109] highlights the efficacy of polyphenylene sulfide (PPS)-based solid-state separators (PPS-SSS) in addressing polysulfide shuttling and interfacial instability in sodium-ion batteries (SIBs) using FeS@C anodes. The PE-PPS-CSSS composite separator, featuring a TCBQ-functionalized PPS matrix and polyethylene (PE) protective layer, chemically immobilizes polysulfides via nucleophilic substitution (evidenced by ATR-FTIR/XPS), while enabling selective Na^+^ transport with low diffusion barriers (0.20 eV) and high ionic conductivity (10^−4^ S cm^−1^). The dense PPS structure (<9% porosity post cycling) ensures mechanical robustness (>35 MPa) and electrochemical stability (EPW > 5.1 V), synergizing with FeS@C’s high capacity (360 mAh g^−1^, 90% initial Coulombic efficiency) to deliver exceptional cycling performance—full cells with Na_3_V_2_(PO_4_)_3_ cathodes retain 81% capacity after 600 cycles at 1C, outperforming conventional PE separators (58.9%). Combined with cost-effective dry-process fabrication and scalable PTFE supply chain optimization, this work establishes PPS-SSS as a transformative solution for high-energy, durable SIBs in grid-scale storage.

An aqueous Zn-Mn battery study [106] employed a sandwich-structured polyphenylene sulfide (PPS) solid-state separator (Paper-PPS-PE) to synergize with Zn-In alloyed solvent-free electrodes, addressing critical challenges in aqueous Zn-Mn batteries. The PPS interlayer (~90 μm) ensures mechanical robustness (>35 MPa) to resist Zn dendrite penetration, while its low porosity (<9%) minimizes polysulfide shuttling and parasitic reactions. Coupled with a hydrophilic polyethylene (PE) protective layer and cellulose paper cathode interface, the composite separator enhances electrolyte wettability and enables uniform Zn^2+^ flux (ionic conductivity > 10^−4^ S cm^−1^), reducing polarization by 40% compared to conventional membranes. This architecture stabilizes the Zn/electrolyte interface, suppressing hydrogen evolution (overpotential increased by 50 mV) and enabling dendrite-free plating even at 5 mA cm^−2^. Full cells with high-loading γ-MnO_2_ cathodes (10 mg cm^−2^) achieve 380 cycles (99% CE), while 2.4 Ah pouch cells retain 80% capacity after 200 cycles at 5 mAh cm^−2^—outperforming standard separators. The scalable dry-process fabrication of PPS separators, combined with cost-effective PTFE supply chains, underscores their viability for grid-scale Zn-ion battery deployment.

A dry-process rolled poly(phenylene sulfide) (PPS) solid-state separator outperforms conventional wet-process porous counterparts in terms of economic viability. The wet-process separator incurs a total production cost of USD 1.51/m^2^, with USD 0.12/m^2^ allocated to material cost (pure PPS resin USD 9230/ton, 50% porosity, 20 μm thickness) and USD 1.38/m^2^ to processing cost, which is further divided into solvent recycling losses of USD 0.92/m^2^, energy-intensive phase separation of USD 0.31/m^2^, and environmental treatment of USD 0.15/m^2^. Conversely, the dry-process separator achieves a total cost of USD 0.64/m^2^, with USD 0.25/m^2^ for material cost (94 wt% PPS, 6 wt% fibrillated PTFE) and USD 0.38/m^2^ for processing cost, including USD 0.23/m^2^ for PTFE fibrillation energy consumption and USD 0.15/m^2^ for rolling operation. By eliminating solvent reliance and streamlining manufacturing steps, the dry process reduces processing costs by over 70%, resulting in total costs at merely 45% of the wet-process baseline. Although the lower porosity (3–6%) of dry-process separators may marginally attenuate ionic conductivity, they significantly bolster assembly reliability and safety in lithium-ion batteries. Future cost reductions for PPS and PTFE are expected. This would further lower material costs to USD 0.20/m^2^ and drive total separator costs below USD 0.46/m^2^, positioning dry-process PPS technology as a transformative solution for scalable, high-safety energy storage systems.

## 5. Summary and Outlook

With the escalating demand for elevated energy densities in lithium-ion battery systems, improving electrochemical safety has emerged as a critical research priority in energy storage technology development. As a critical component for ensuring battery safety, the further development of battery separators is of paramount importance. An optimal battery separator membrane must exhibit the following essential attributes: sustained chemical inertness and electrochemical durability to guarantee operational longevity; excellent ion transport capability to improve electrochemical performance; sufficient mechanical strength and good thermal stability to enhance battery safety. Additionally, simple preparation processes and low costs are also key factors determining whether the separator can achieve large-scale production and application.

This article comprehensively reviews the current preparation processes used for producing PPS separators, as well as the latest research progress on various types of PPS separators in high-energy-density lithium metal batteries and lithium metal-free batteries, summarizing their physical and electrochemical properties. For example, high-porosity fiber separators prepared via spinning processes and pore-free separators prepared through dry-film forming processes both demonstrate unique advantages. Additionally, PPS separators combined with other polymers, fibers, or commercial separators exhibit excellent physical and electrochemical performance, particularly in preventing battery thermal runaway and suppressing lithium dendrite growth during cycling. These studies provide important theoretical and technical support for the development of high-performance, high-safety battery separators.

As mentioned above, although PPS separators have many potential advantages in lithium-ion battery applications, research in this field is still in its early stages, and several issues need to be addressed before PPS separators can be commercialized. Currently, the preparation processes for both PPS separators and composite separators have significant shortcomings. For example, the production of nonwoven separators using spinning processes is not only costly and inefficient but also requires the extensive use of solvents in the subsequent preparation of composite separators, which not only increases production costs but also has a negative environmental impact. Additionally, processes such as TIPS and particle leaching suffer from high energy consumption and excessive solvent use. Consequently, the refinement of conventional manufacturing protocols and development of novel synthesis methodologies to minimize production expenditures while maintaining optimal performance-cost equilibrium has emerged as a pivotal research focus. On the other hand, the thickness of current PPS-based separators generally exceeds 25 μm and can even reach over 50 μm. Excessively thick separators lead to increased internal resistance, higher costs, and reduced energy density, severely limiting their practical application.

To overcome thickness constraints while preserving mechanical integrity, ultrathin PPS separators (<15 μm) can be engineered through dry-process optimization. This involves refining ultra-low clearance kneaders to achieve efficient and synchronized uniform powder shear forces, enabling thorough integration of crystalline PPS particles with nanoscale PTFE fibrils (3–5 wt%) to construct stress-dissipative networks. During hot-rolling, the introduction of trace amounts of deformable polymers (e.g., nylon) facilitates crack self-healing under thermal stress. For multifunctional enhancement, a 100–200 nm Al_2_O_3_/ZrO_2_ bilayer coating deposited via atomic layer deposition (ALD), gravure printing, electrostatic spraying, and related processes synergistically improves dendrite suppression (Young’s modulus > 15 GPa) and thermal stability. Concurrently, sulfur-anchored graphene nanochannels (10–20 nm width) integrated during PPS film formation establish rapid Li^+^/Na^+^ transport pathways, achieving ionic conductivity exceeding 1 mS·cm^−1^. Scalable manufacturing can be realized through laser-assisted crystallinity modulation in roll-to-roll processes, enabling thickness gradients (12–25 μm) tailored to electrode surface roughness. These innovations, grounded in existing PPS dry-film technologies, demonstrate potential for significantly advancing the commercialization of high-safety lithium metal batteries with ultra-high energy densities (500–700 Wh kg^−1^).

## Figures and Tables

**Table 1:** Summary of key parameters of various PPS separators.

Separator	Process	Drying Temperature (°C)	Casting Method (Mass Ratio)	Thickness (μm)	Porosity (%)	Electrolyte Uptake (%)	Ionic Conductivity (mS⋅cm^−1^)	Reference Number
***Porous PPS separators***								
PPS/SiO_2_	Particle leaching			50	**High**			[96]
PPS1-81	TIPS	50 (12 h)		29	73.5	409	1.69	[97]
PPS2-81	TIPS	50 (12 h)		28	69.7	384	1.61	[97]
PPSC	melt-blown spinning	50 (24 h)	SiO_2_:PVDF-HFP:acetone:DMF = 3:5:45:5	114	57.3	230.1	1.02	[98]
CLN/PPS	melt-blown spinning	60 (8 h)	PEI:PVDF-HFP:acetone:DMF = 1:15:120:30	95	65	197	0.52	[99]
CS@4	melt-blown spinning	60 (12 h)	PAMPAM:PVDF-HFP: acetone:DMF = 1:10:60:40	51	85	236	0.92	[100]
3D-30:1	melt-blown spinning	60 (12 h)	PVDF-HFP:DMF = 1:10+ silica-based fluid:methanol = 0.33:2	63	72.1	153.6	0.51	[101]
3D-10:1	melt-blown spinning	60 (12 h)	PVDF-HFP:DMF = 1:10+ silica-based fluid:methanol = 0.1:2	66	78.6	202.5	0.92	[101]
3D-10:3	melt-blown spinning	60 (12 h)	PVDF-HFP:DMF = 1:10+ silica-based fluid:methanol = 0.3:2	70	68.4	172.9	0.65	[101]
ANFs/PPS	melt-blown spinning	60 (24 h)	PPS:ANFs = 100:15	50	65.9	240.7	1.43	[102]
CFs/PPS	melt-blown spinning	80 (24 h)	PPS:CFs = 1:1		61.1	259.6	1.26	[103]
PPS-mGNFs	sea-island spinning	100 (8 h)	PPS: mGNFs = 100:20	34	65.9	253	1.43	[104]
***Non-porous PPS separators***								
PPS-CSSS	dry-film forming process	100 (12 h)	Crystalline PPS: fibrillated PTFE = 94:6	30	6%	10%	0.3	[39]
PPS-SSS	dry-film forming process	100 (12 h)	Crystalline PPS: fibrillated PTFE = 94:6	18	3%	5%	0.2	[105]

## Data Availability

No new data were created or analyzed in this study.
